# Supplementary material for: Seasonal variations in photoperiod affect hepatic metabolism of medaka (Oryzias latipes)
Source: FEBS Open Bio. 2021 Feb 28;11(4):1029–40. doi: 10.1002/2211-5463.13095 (PMC8016123; doi:10.1002/2211-5463.13095)
Supplement: Supplementary file 1 — Fig S1. Diurnal changes in metabolites related to glycolysis. [file FEB4-11-1029-s001.docx]

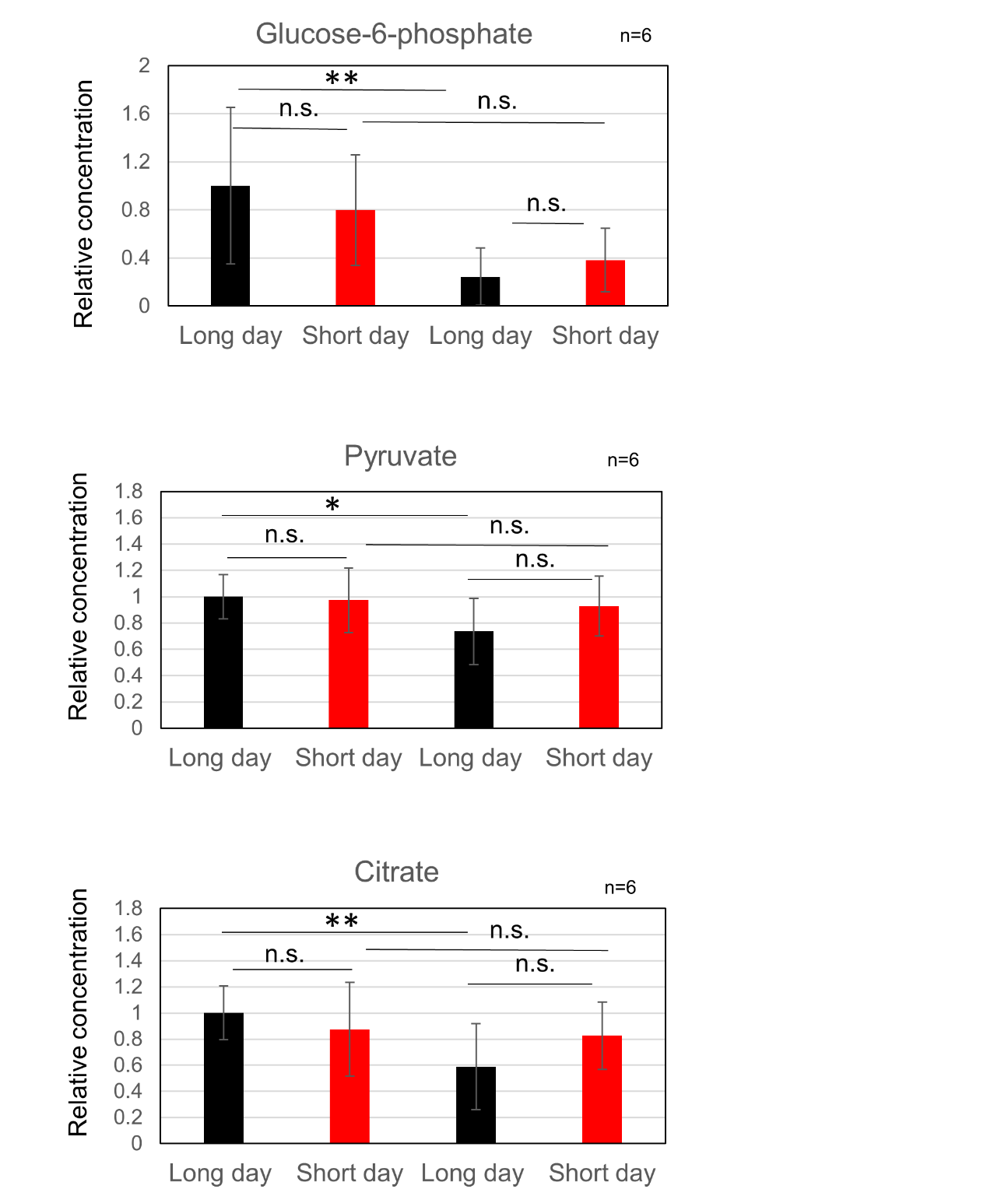


Fig S1. Diurnal changes in metabolites related to glycolysis

The long day group was illuminated from 7:00 a.m. to 9:00 p.m. The short day group was illuminated from 9:00 a.m. to 7:00 p.m.

Liver samples were collected at 2:00 p.m. (day) or at 2:00 a.m. (night).

Glucose-6-Phosphate Assay Kit (Colorimetric) (ab83426), EnzyChrom Pyruvate Assay Kit (BioAssay Systems Hayward, CA 94545, U. S. A.) and EnzyChrom Citrate Assay Kit (BioAssay Systems Hayward, CA 94545, U. S. A.) were used to measure the concentration of metabolites.

The error bars indicate SD (n=6). * indicates p<0.05, and ** indicates p<0.01 (Steel-Dwass).
