# Supplementary material for: Seasonal variations in photoperiod affect hepatic metabolism of medaka (Oryzias latipes)
Source: FEBS Open Bio. 2021 Feb 28;11(4):1029–40. doi: 10.1002/2211-5463.13095 (PMC8016123; doi:10.1002/2211-5463.13095)
Supplement: Supplementary file 2 — Table S1. Real‐time RT‐PCR primer sequence. [file FEB4-11-1029-s002.docx]

**Supplementary Table 1. Real-time RT-PCR primer sequence**

| *gapdh*　Forward | 5′-GACCTGCTTTCACCTCTAAA-3′ |
| --- | --- |
| *gapdh*　Reverse | 5′-TTGTGGGTGGAGTCAATCTT-3′ |
| *ald*　Forward | 5′-AGAAGGAGAATGGAAAGGCA -3′ |
| *ald*　Reverse | 5′- AGCATGGTTAGCCACAAACA -3′ |
| *mdh*　Forward | 5′- AAGGAGGCGTGGTGGAGT-3′ |
| *mdh*　Reverse | 5′- GTTCTTCTCAATGCCGCTCT-3′ |
| *fumarase*　Forward | 5′- AGATTGCCAACGACATCCGT-3′ |
| *fumarase*　Reverse | 5′- CACTGGGTGGGGTTCACTTT-3′ |
| *cs*　Forward | 5′-ACGTTGTCACCATGCTGGAT -3′ |
| *cs*　Reverse | 5′- TGTGATCGCAGCACTGAACT-3′ |
| *cpt1a* Forward | 5′- ATGTCTACCTCCGTGGACGA -3′ |
| *cpt1a* Reverse | 5′- CAAGTTTGGCCTCTCCTTTG -3′ |
| *pparα*　Forward | 5′- AGGGTTGCAAGGGTTTCTTT -3′ |
| *pparα*　Reverse | 5′- AGCTTCAGCTTCTCCGACTG -3′ |
| *acox1* Forward | 5′- ACAAGAGCATGGTCACAGGC-3′ |
| *acox1*Reverse | 5′- GGCAGCCATTTGCTCATCTG-3′ |
| *ef1α* Forward | 5′- AACACTCCTTGAAGCTCTTG-3′ |
| *ef1α* Reverse | 5′- GACAGGGACAGTTCCAATAC-3′ |
